# Hemodynamic analysis for stenosis microfluidic model of thrombosis with refined computational fluid dynamics simulation

**DOI:** 10.1038/s41598-021-86310-2

**Published:** 2021-03-25

**Authors:** Yunduo Charles Zhao, Parham Vatankhah, Tiffany Goh, Rhys Michelis, Kiarash Kyanian, Yingqi Zhang, Zhiyong Li, Lining Arnold Ju

**Affiliations:** 1 School of Biomedical Engineering, Faculty of Engineering, The University of Sydney, Darlington, NSW 2008 Australia; 2 Charles Perkins Centre, The University of Sydney, Camperdown, NSW 2006 Australia; 3 Heart Research Institute, Newtown, NSW 2042 Australia; 4 School of Chemical and Biomolecular Engineering, Faculty of Engineering, The University of Sydney, Darlington, NSW 2008 Australia; 5 School of Mechanical, Medical and Process Engineering, Queensland University of Technology, Brisbane, 4000 Australia

**Keywords:** Biomedical engineering, Lab-on-a-chip

## Abstract

Disturbed blood flow has been increasingly recognized for its critical role in platelet aggregation and thrombosis. Microfluidics with hump shaped contractions have been developed to mimic microvascular stenosis and recapitulate the prothrombotic effect of flow disturbance. However the physical determinants of microfluidic hemodynamics are not completely defined. Here, we report a refined computational fluid dynamics (CFD) simulation approach to map the shear rate (*γ*) and wall shear stress (*τ*) distribution in the stenotic region at high accuracy. Using ultra-fine meshing with sensitivity verification, our CFD results show that the stenosis level (*S*) is dominant over the bulk shear rate (*γ*_0_) and contraction angle (*α*) in determining *γ* and *τ* distribution at stenosis. In contrast, *α* plays a significant role in governing the shear rate gradient (*γ*^′^) distribution while it exhibits subtle effects on the peak *γ*. To investigate the viscosity effect, we employ a Generalized Power-Law model to simulate blood flow as a non-Newtonian fluid, showing negligible difference in the *γ* distribution when compared with Newtonian simulation with water medium. Together, our refined CFD method represents a comprehensive approach to examine microfluidic hemodynamics in three dimensions and guide microfabrication designs. Combining this with hematological experiments promises to advance understandings of the rheological effect in thrombosis and platelet mechanobiology.

## Introduction

Thrombotic diseases have become the leading cause of modern mortality^1,2^. Thrombi, also known as blood clots, mainly consist of aggregated platelets. They become dangerous when they grow large and occlude blood vessels in the heart, brain and peripheral vascularized organs, leading to heart attack, stroke and deep vein thrombosis respectively^2^. While it is well known that platelet adhesion, activation, and subsequent aggregation play a central role in thrombosis, the interplay of biochemical and biomechanical factors regulating platelet thrombosis remains incompletely understood^3–5^.

Recent studies have observed the association between enhanced platelet aggregation and blood flow disturbance^3,6^. In vivo, blood flow within vessels exerts hemodynamic forces on both the vessel surface in the form of wall shear stress (WSS; *τ*), as well as blood components such as red blood cells (RBC), white blood cells and platelets in the form of shear rate (*γ*). *τ* is the tangential force on the vessel or channel wall due to friction, while *γ* describes the shear effect that the interstitial fluid experiences during flow^7^. It has been recognized that a high shear rate and its gradient may activate mechanosensitive proteins such as von Willebrand factor (VWF)^8,9^ and platelet glycoprotein Ib receptor^10–12^, leading to subsequent platelet activation and aggregation^7,13^. Nevertheless, the relationship between the microfluidic boundary conditions (*γ*_0_, *S*, *α*,) and hemodynamic parameters of flow disturbance, such as the peak shear rate (*γ*_max_), peak WSS (*τ*_max_) and shear rate gradient (*γ*^′^) at the stenotic (narrowing) region, remains incompletely defined^5,14,15^. Furthermore, it has been hypothesized that biomechanical platelet aggregation is not simply attributed to elevated shear and elongational force at the narrowing apex alone^4^, but the entire ‘shear history’ where the platelet experiences accumulated mechanical stimuli and concomitant cellular interactions^15,16^. Thus, it is important to map the hemodynamic distribution at both the pre- and post-stenosis regions^5^.

**Figure 1:**
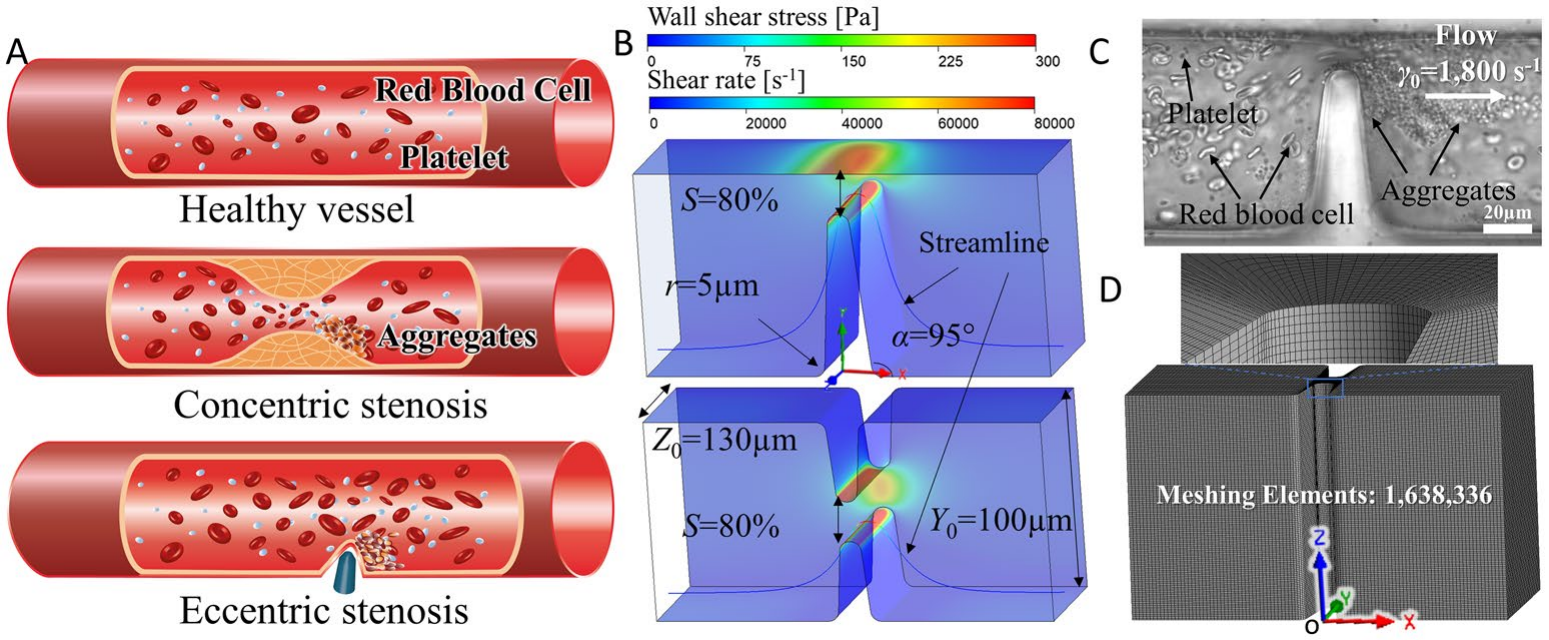
Pathological and physiological blood vessel stenoses and the mimicking microfluidic models. (**A**) Blood flow in a healthy vessel without stenosis (*top*), in a vessel with concentric stenosis due to atherosclerotic plaque (*middle*) and a vessel with eccentric stenosis due to medical device insertion (*bottom*). (**B**) CFD contour maps in the eccentric (*top*) and concentric (*bottom*) stenosis microfluidic channels. Note that the channel walls were colored to display the WSS distribution; the representative streamline of a platelet trajectory was colored to display the shear rate *γ* distribution. (**C**) Differential interference contrast microscopy image of biomechanical platelet aggregation in an eccentric stenosis microfluidic channel after whole blood perfusion at *γ*_0_ = 1,800 s^−1^, mimicking the physiological shear rate in arteries and arterioles^7^. To visualize platelet aggregates in better clarity, the microchannel was washed with Tyrode’s buffer after whole blood perfusion. Note that platelets aggregate at the downstream face of the stenosis. Scale bar = 20 µm. (D) ANSYS finite volume meshing scheme for the concentric stenosis model as in panel C. The entire microfluidic channel was meshed into 1,638,336 hexahedral elements for CFD analysis. The coordinate origin is located at the center of the front bottom edge. Note that for illustration purpose, a coarse mesh is shown in the figure.

In a healthy patent blood vessel (Fig. 1A, *top*), the blood flow pattern is laminar and steady, yielding a constant velocity profile, *γ* and *τ* distribution. However, when a vessel is narrowed concentrically from all directions (e.g. atherosclerotic plaque formation; Fig. 1A, *middle*)^30^, or eccentrically narrowed from one direction (e.g. medical device insertion; Fig. 1A, *bottom*)^31,32^, the *τ* and *γ*_0_ become elevated at the stenosis with blood flow acceleration (Table 1). To mimic the pathological microvascular conditions, stenosis microfluidic models of thrombosis have been designed with a default width of *Y*_0_ = 100 µm, height of *Z*_0_ = 130 µm, *α* = 85° and *S* = 80% (Fig. 1B)^13,33^. It is worth noting that the flow profile within such microchannels is considered two-dimensional (2D), as the geometry and flow vary only in the x–y plane at the stenotic region (Fig. 1B)^7,13,34^. Previous studies have justified these stenosis microfluidic models of thrombosis which have been well characterized and validated experimentally^6,7,13,16,38,40^, demonstrating the shear rate gradient effects on platelet aggregation in blood flow disturbance. The advantages of such microfluidic approaches enable direct visualization of biomechanical platelet aggregation at the downstream face of stenosis (Fig. 1C). Nevertheless, the detailed rheological mechanisms, or specifically the exact *τ*, *γ* and *γ*^′^ thresholds that trigger such biomechanical platelet aggregation are incompletely understood. In this context, CFD simulation represents the first step in revealing the hemodynamic profile within disturbed blood flow and correlating with experimental results of thrombotic response.

**Table 1:** Physiological and pathological shear rate ranges of in vivo circulating blood.

Vascular conditions	Vessel types	WSS τ (dyne/cm^2^)	Bulk shear rate *γ*_0_ (s^−1^)
Healthy vessel	Arteries	5–36^17–19^	300–800^18,20^
	Veins	1–6^17,21,22^	15–200^18,20^
	Microvessels	20–80^21^	450–1800^18,20^
Concentric stenosis	Atherosclerotic plaque	36–6,000^23,24^	800–10,000^18,20,25^
Eccentric stenosis	Occlusive thrombus	36–450^17^	5,000–400,000^25^
	Coronary stent	0–40^17,26^	0–11,000^27^
	Catheter insertion	0–20,000^17,28^	> 10,000^29^

CFD is the most popular computational method to model hemodynamic parameters i.e. *τ*, *γ* and *γ*^′^ and simulate microfluidic outcomes before experiments are done^7,15^. However, rarely do the CFD practices nowadays thoroughly show the mesh sensitivity verification, or systematically benchmark the effects of the microfluidic boundary conditions on the hemodynamic outcome with certain control variables i.e. *γ*_0_, *S*, *α* and fluid medium (Table 2). The caveat is that the CFD solutions may have some extents of error with coarse mesh sizes. Besides, although the non-Newtonian property of blood has significant impacts on the viscosity, its influence in the disturbed flow region remains poorly characterized. Hereby, we present an ultra-fine CFD study to map hemodynamic profiles for stenosis microfluidic models of thrombosis, and address the above concerns with mesh sensitivity verification and analytical validation.

**Table 2:** Working fluids modeled and physical properties.

Working fluid	Density *ρ* (kg m^−3^)	Viscosity *µ* (Pa s)
Water	998	0.001003
Blood (Newtonian)	1,060	0.003450
Blood (non-Newtonian)	1,060	GPL model

## Methods

### Stenosis microfluidic models of thrombosis

Microfluidics is a miniaturized approach that has significant advantages in controling hemodynamic parameters, ligand presentation and agonist stimulation at the micro-scale^13,35–37^. Microfluidic channels with hump-like contractions have been developed and utilized to mimic microvessel stenosis and examine the prothrombotic effect of flow disturbance through which whole blood or washed platelets are perfused (Fig. 1C)^6,7,13,38–41^.

### Numerical formulation and governing equations

The commercially available software ANSYS FLUENT version 2020 R1 is utilized to computationally simulate the flow profile. The flow was assumed as steady, laminar, and incompressible. Under these assumptions, the fluid can be described using the continuity Eq. (1) and Cauchy momentum Eq. (2) as follows:

1 $$\nabla \cdot u = 0$$

2 $$\rho \left( {u \cdot \nabla u} \right) = - \nabla P + \nabla \cdot \left[ {\mu \left\{ {\left( {\nabla u} \right) + \left( {\nabla u} \right)^{T} } \right\}} \right]$$

where *u* is the 3D velocity vector (m s^−1^), *P* is the pressure (Pa), $\mu$ is the dynamic viscosity (Pa s) and *ρ* is the density (kg m^−3^). This can be simplified to the well-known Navier–Stokes equation for the case of Newtonian fluids (fluids with constant dynamic viscosity):

3 $$\rho \left( {u \cdot \nabla u} \right) = - \nabla P + \mu \nabla^{2} u$$

In ANSYS FLUENT, to assess the viscosity effect we simulated 3 working mediums: water, blood as a Newtonian fluid, and blood as a non-Newtonian fluid under the Generalized Power-Law (GPL) viscosity model (Table 2).

Numerous non-Newtonian models have been used throughout literature for simulating blood flow: Carreau^42^, Modified Cross Law (Carreau-Yasuda)^43^, Power-Law (PL)^44^, non-Newtonian Power-Law^44^, Generalized Power-Law^45^, Casson^46^ and Walburn-Schneck Law^47^ are among the well-recognized models^48^. In this study, the GPL model is utilized due to its accuracy in calculating the shear rate *γ*^48^, which is calculated from:

4 $$\mu = K\gamma^{n - 1}$$

where *K* is the consistency index, and *n* is the Power-Law index. The two parameters are calculated from^45^:

5 $$K = \mu_{\infty } + \delta \mu \,exp\left( { - \left( {1 + \frac{\gamma }{a}} \right)exp\left( {\frac{ - b}{\gamma }} \right)} \right)$$

6 $$n = n_{\infty } - \delta n \,exp\left( { - \left( {1 + \frac{\gamma }{c}} \right)exp\left( {\frac{ - d}{\gamma }} \right)} \right)$$

where *µ*_*∞*_ = 0.00345 Pa s, *n*_*∞*_ = 1.0, *δµ* = 0.25, *δn* = 0.45, *a* = 50, *b* = 3, *c* = 50 and *d* = 4^45^.

### Geometrical properties, boundary conditions and Reynolds number

A schematic diagram of the microfluidic channel is illustrated in Fig. 1B. By default, the axial length is *X*_0_ = 200 µm, the width is *Y*_0_ = 100 µm and the height of the cross-section is *Z*_0_ = 130 µm. A zero-gauge pressure boundary condition is applied at the inlet of the microchannel (Fig. 1B). A no-slip boundary condition is applied to the walls. Finally, a boundary condition of the experimentally implemented flow rate *Q* (µL min^−1^) is implemented at the outlet of the flow region (Fig. 1B,C). The flow rate calculation is defined as^49^:

7 $$Q = \frac{{0.12AD_{h} \gamma_{0} }}{\lambda }$$

where $\gamma_{0}$ (s^−1^) is the bulk shear rate, *A* (µm^2^) is the cross-sectional area, *D*_h_ (m) is the hydraulic diameter, and, *λ* is the shape factor of the microfluidic cross-section^49^. *A*, *D*_h_ and $\lambda$ are calculated by the following equations:

8 $$A = Y_{0} Z_{0}$$

9 $$D_{h} = \frac{2A}{{Y_{0} + Z_{0} }}$$

10 $$\lambda = \frac{24}{{\left[ {\left( {1 - 0.351Y_{0} /Z_{0} } \right)\left( {1 + Y_{0} /Z_{0} } \right)} \right]^{2} }}$$

The above also allows for the calculation of the Reynolds number, a dimensionless group used to indicate whether fluid flow in a channel is characterized as laminar or turbulent. The Reynolds number for a Newtonian fluid is given by:

11 $$Re = \frac{{D_{h} \rho^{2} Q}}{\mu A}$$

Substituting *Q* from Eq. (7) into Eq. (11) yields the following expression of the Reynolds number for this particular investigation:

12 $$Re = \frac{{0.12D_{h}^{2} \rho^{2} \gamma_{o} }}{\lambda \mu }$$

Using *γ*_0_ = 150 and 3,000 s^−1^ for Eq. (12), the Reynolds number for this study is found to range from 0.04 to 0.9. Unlike the turbulent state of blood flow in the stenotic region of the common carotid arteries with large diameters shown in some studies^50–52^, a Reynolds number with such low magnitude indicates a laminar flow regime, as flow typically does not transition into a turbulent regime before the critical point of *Re* = 2,300^53^, validating our laminar flow assumption.

Furthermore, our steady flow assumption stems from the Windkessel effect which dampens the pulsatile flow changes far away from the heart, leading to steady flow in the capillary region^54,55^. This steady assumption is further validated by the Womersley number which is a dimensionless group to evaluate the pulsatile flow effect in relation to oscillation frequency and viscosity^56^.

13 $$Wo = \frac{D}{2}\sqrt {\frac{\rho \omega }{\mu }}$$

14 $$\omega = 2\pi f$$

where *D* is the diameter of the vessel (m); *f* and *ω* are the oscillation and angular frequency (both Hz) respectively. We selected *f* = 2 Hz and *D* = 130 µm, corresponding to a heart rate of 120 beats per minute to validate our assumption at even extreme conditions. The calculated Womersley number of 0.128 is much less than the critical point of *Wo* = 1^56^, validating our steady flow assumption.

### Stenosis microfluidic control variables

**Figure 2:**
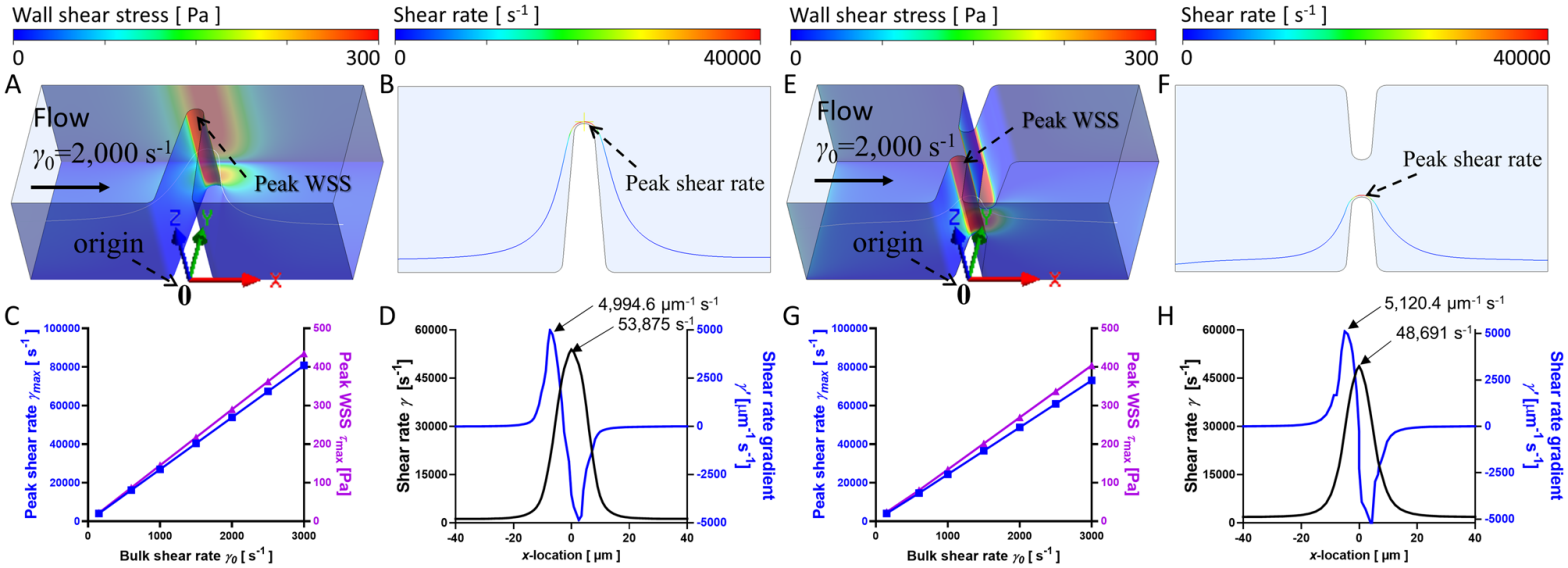
CFD simulated shear rate, WSS and shear rate gradient plot for eccentric (**A**–**D**) and concentric (**E**–**H**) stenosis microfluidics under various bulk shear rates. (**A** and **E**) The cross-section WSS view of the eccentric (**A**) and concentric (**E**) stenoses microfluidic for *γ*_0_ = 2,000 s^−1^. Note that the WSS occurs at the middle of stenotic region. (**B** and **F**) The streamline of blood flow in the eccentric and concentric stenosis microfluidic channel for *γ*_0_ = 2,000 s^−1^. The representative streamline of a platelet trajectory was colored by shear rate values. Note that the peak shear rate *γ*_max_ occurs at the stenosis apex. (**C** and **G**) The peak shear rate *γ*_max_ and peak WSS *τ*_max_ linearly correlated with the input bulk shear rate *γ*_0_ for both eccentric and concentric stenosis microfluidics respectively. (**D** and **H**) The shear rate and shear rate gradient history of a single platelet particle trajectory 30 μm from the bottom wall and 1 μm from the symmetric geometry surface at *γ*_0_ = 2000 s^−1^ for eccentric and concentric stenosis microfluidic channels. Note that the *γ*_max_ ≈50,000 s^−1^ and *γ*^’^_max_ ≈5,000 µm^−1^ s^−1^ for eccentric and concentric stenosis respectively.

**Figure 3:**
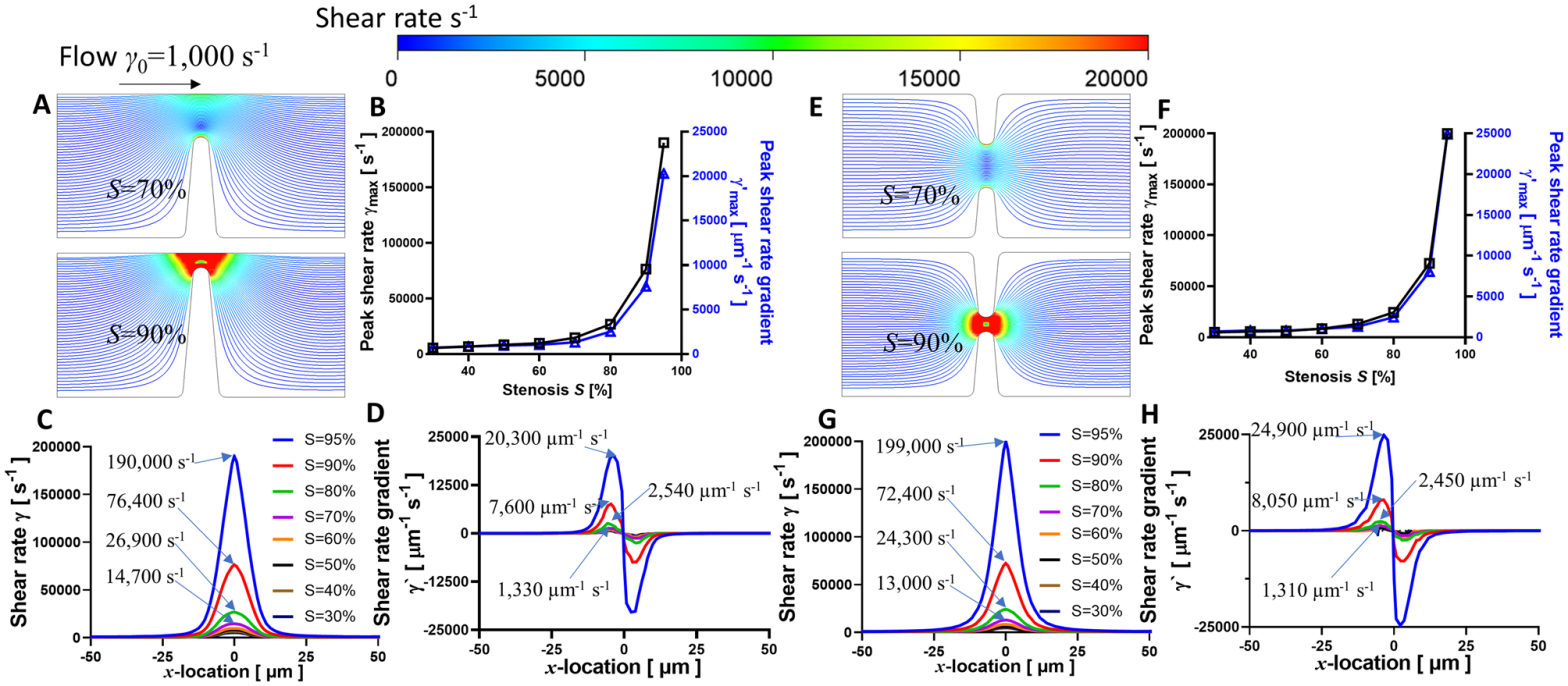
CFD simulated shear rates and shear rate gradients in eccentric (**A**–**D**) and concentric (**E**–**H**) stenoses geometry under various stenosis level. (**A** and **E**) The streamlines of blood flow in the eccentric and concentric stenosis microfluidic channels for S = 70% (*top*) and 90% (*bottom*) respectively. The representative streamline of a platelet trajectory was colored by shear rate values. (**B** and **F**) Peak shear rate *γ*_max_ exponentially correlates with the input stenosis level *S* for both eccentric and concentric stenoses respectively. Note that the concentric *γ*_max_ is higher than that of eccentric stenosis channel. The shear rate *γ* (**C** and **G**) and shear rate gradient *γ*^′^ (**D** and **H**) distribution is plotted along a sample streamline 1 µm above the stenosis apex spanning the shear acceleration (*x* = −100 to 0 µm) and deceleration (*x* = 0–100 µm) zones. Note that *γ*_max_ and *γ*^’^_max_ occur at the same location as in Fig. 2.

**Figure 4:**
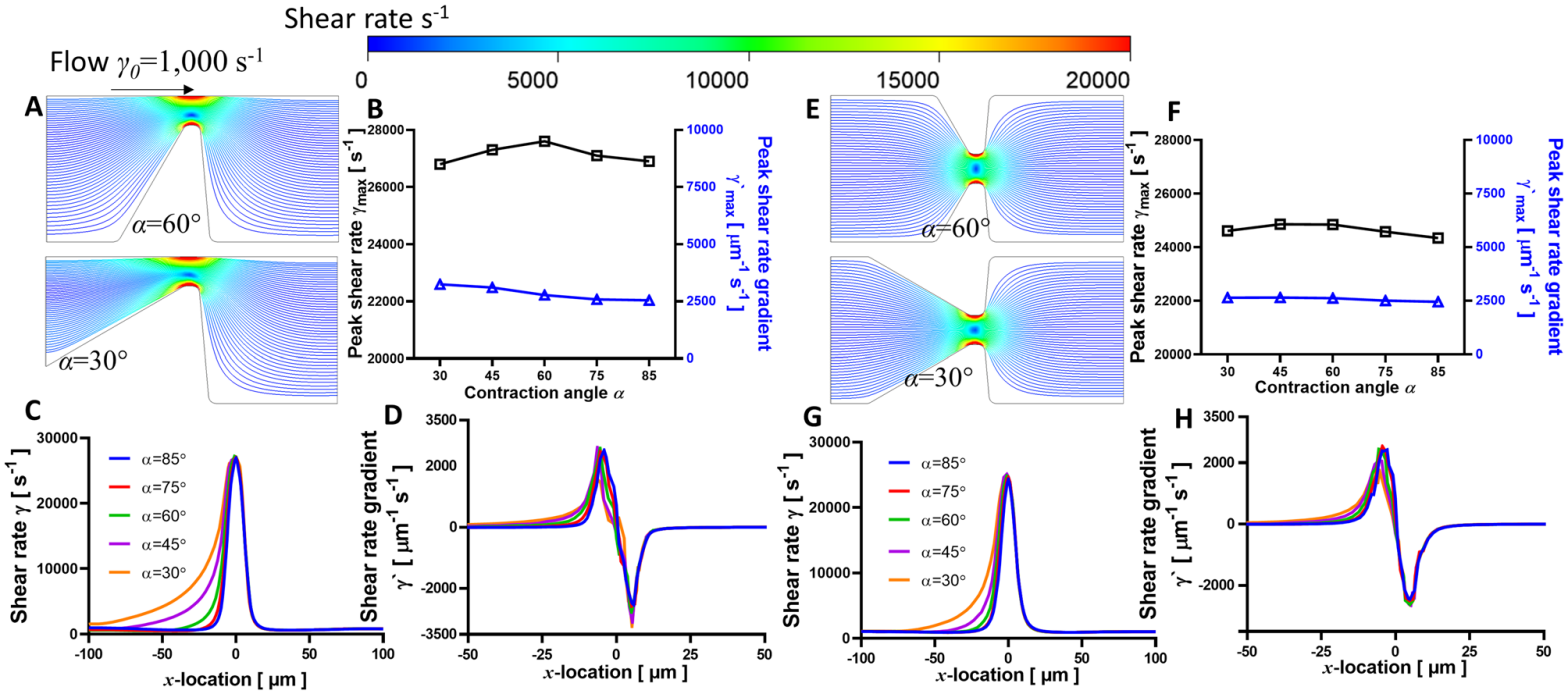
CFD simulated shear rates and shear rate gradients in eccentric (**A**–**D**) and concentric (**E**–**H**) stenoses geometry under various contraction angles. (**A** and **E**) The streamlines of blood flow in the eccentric stenosis and concentric stenosis microfluidic channel for *α* = 60° (*top*) and 30° (*bottom*) respectively. The representative streamline of a platelet trajectory was colored by shear rate values. (**B** and **F**) Peak shear rate *γ*_max_ slightly changes correlated to the contraction angle for both eccentric and concentric stenoses geometries. Note that the eccentric stenosis upstream shear rate is higher than that of the concentric stenosis channel. The shear rate *γ* (**C** and **G**) and shear rate gradient *γ*^′^ (**D** and **H**) distribution is plotted along a sample streamline 1 µm above stenosis apex spanning the shear acceleration (*x* = −100 to 0 µm) and deceleration (*x* = 0–100 µm) zones. Note that the shear rates in the post-stenosis zone remain unchanged with respect to *α*, while the upstream of the pre-stenosis area displays faster acceleration.

**Figure 5:**
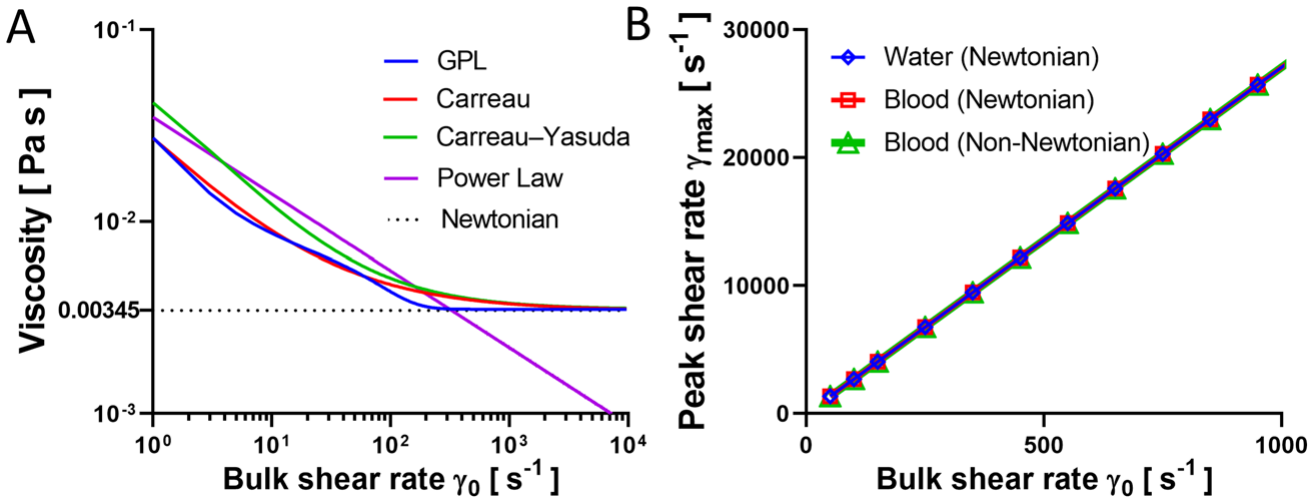
Benchmarks of viscosity models and flow medium effects. (**A**) Relationship between bulk shear rate *γ*_0_ and viscosity for the different viscosity models. Note that the viscosity for most models first decreases then approaches to constant 0.00345 Pa s at *γ*_0_ > 500 s^−1^. (**B**) Relationship between peak shear rate and viscosity for water (blue), Newtonian blood (red) and non-Newtonian blood (green). The peak shear rates *γ*_max_ are indifferent for all cases.

To define the physical determinants of hemodynamic profiles, four studies were conducted with microfluidic control variables as defined in Table 3: Study (1) the bulk (wall) shear rate *γ*_0_ = 150–3000 s^−1^ by adjusting *Q* = 1.84–36.77 µL min^−1^ as calculated with Eq. (7) (Fig. 2); Study (2) the stenosis level *S* = 30—95% are assessed under *γ*_0_ = 1,000 s^−1^ (Fig. 3); Study (3) the contraction angle *α* = 30—85° are assessed under *γ*_0_ = 1,000 s^−1^ (Fig. 4); Study (4) benchmark the viscosity effect on water, blood as Newtonian fluid, and blood as non-Newtonian fluid (Fig. 5).

**Table 3:** Control variable selection.

Control variables	Test values	Default values
Bulk shear rate *γ*_0_ (s^−1^)	*γ*_0_ = 150, 600, 1000, 1500, 2000, 2500, 3000 s^−1^	*α* = 85°, *S* = 80%, Blood
Stenosis level *S* (%)	*S* = 30, 40, 50, 60, 70, 80, 90, 95%	*γ*_0_ = 1,000 s^−1^, *α* = 85°, Blood
Contraction angle *α* (°)	*α* = 30, 45, 60, 75, 85°	*γ*_0_ = 1,000 s^−1^, *S* = 80%, Blood
Fluid medium	Water; Blood; non-Newtonian Blood; *γ*_0_ = 50–1,050 s^−1^	*S* = 80%, *α* = 85°

By default, the shear rate and velocity profile are simulated with a streamline sampled as the single platelet trajectory passing 1 µm (half of the diameter of a single platelet) above the stenosis apex, and *z* = 30 µm from the bottom (Fig. 1B).

In addition, we set up an ultra-fine CFD mesh with approximately 2,000,000 elements (Fig. 1D and Supplementary Fig. S1) for all the studies to achieve high accuracy.

## Results

### Bulk shear rate impact on WSS and shear rate distribution at stenosis

It has been well recognized that platelets activate and aggregate in response to flow disturbance^57–59^. In microcirculation, blood flow is considered laminar and steady as the change in geometry does not transfer the flow into a turbulent regime. Here we investigated a wide range of *γ*_0_ = 150–3,000 s^−1^ and the effects on the *τ* and *γ* distribution using our refined CFD method. Although *τ*_max_ is similar on both eccentric (Fig. 2A) and concentric (Fig. 2E) stenosis microfluidic wall with 7% difference, a higher *τ* distribution is observed at the ceiling of the eccentric stenosis (compare Fig. 2A vs. E), which could lead to higher probabilities of vessel damage^48^.

Then we compared a sample platelet trajectory streamline across the stenotic region (Fig. 2B,F), displaying 10% higher *γ*_max_ in the eccentric stenosis than the concentric stenosis. Interestingly, as *γ*_0_ increases from 150 to 3,000 s^−1^, the *τ*_max_ and *γ*_max_ both increased linearly with respect to *γ*_0_ about 20.0- and 19.4-fold respectively for eccentric stenosis (Fig. 2C). Similarly, the *τ*_max_ and *γ*_max_ increased by 16.7- and 20.0-fold respectively for concentric stenosis (Fig. 2G). Notably, we found eccentric stenosis has larger *γ*_max_ and *γ*^’^_max_ than concentric stenosis (compare *γ*_max_ = 53,875 s^−1^, *γ*^’^_max_ = 25,701 µm^−1^ s^−1^ in Fig. 2D vs. *γ*_max_ = 48,691 s^−1^, *γ*^’^_max_ = 23,295 µm^−1^ s^−1^ in Fig. 2H). Of note, our simulated *τ*_max_ displays strong correlation to *γ*_max_. Thus, we focused on the shear rate *γ* as the examining parameter in the following studies.

### Relation between micro-contraction geometry and hemodynamic profile at stenosis

Flow disturbance caused by vessel lumen constriction has a major prothrombotic effect via platelet mechanosensing^7^. Hereby, we examined *γ* and *γ*^′^ distributions as functions of *S* for degree (Fig. 3) and *α* for topology (Fig. 4) of vessel narrowing. Notably, we observed that *S* plays a significant role in the shear rate acceleration towards *γ*_max_ (Fig. 3A,E). The *γ*_max_ for eccentric and concentric geometries increased 34.6- and 41.0-fold respectively as *S* increased from 30 to 95% (Fig. 3C,G). Similarly, the *γ*^’^_max_ for both geometries increased 28.3- and 35.7-fold respectively (Fig. 3D,E). Notably, we identified an exponential *γ*_max_ increment once the stenosis level exceeded 70% (Fig. 3B,F). Trajectory analyses demonstrated similar increases in *γ* and *γ*^′^ in the *S* > 70% regimes for both concentric (Fig. 3C,D) and eccentric (Fig. 3G,H) stenoses.

In contrast, *α* did not significantly affect the hemodynamics within the stenotic region, and subsequently had little effect on the *γ* and *γ*^′^ distribution (Fig. 4B,F). It is worth noting that with increasing *α*, the upstream face or pre-stenosis area displayed rapid acceleration in *γ* (Fig. 4C,G) and *γ*^′^ (Fig. 4D,H), whilst the post-stenosis downstream area was not affected. Our findings demonstrated that the shear rate profile changes most significantly when *S* is varied, suggesting that (1) although the development of vessel narrowing might be negligible at earlier stages of development, its hemodynamic impacts are rapidly enhanced once severe pro-occlusive conditions are reached; (2) the impact of the stenosis contraction angle is negligible due to its limited influence on *γ*_max_, however, its significant impact on the shear acceleration zone should be further investigated, especially its elongational effects acting on blood cells and plasma proteins^4,9^.

### Hemodynamic benchmarks of viscosity models

The selection criteria for viscosity models on microfluidic simulation remain incompletely standardized. For example, it is fundamental to understand whether the hemodynamic forces experienced by washed platelets in Tyrode’s buffer is the same as those in whole blood^41,60^. Accurate computational modelling of blood is a complicated task due to multiplexed components in whole blood. RBCs consist a large amount, 45% of whole blood by volume or hematocrit^61^. Considering that RBCs have viscoelastic properties, blood exhibits shear-thinning behavior (decreasing viscosity with increasing shear rates), or non-Newtonian feature, at low shear rate *γ* < 1000 s^−1^ (Fig. 5A)^62^. While at high shear rates *γ* > 1000 s^−1^, blood behaves as a Newtonian fluid with a linear relationship between *γ* and *τ*, in another words nearly constant viscosity (Fig. 5A)^62,63^.

To benchmark the viscosity model choice, we mapped the viscosity changes with respect to *γ*_0_ by applying five different constitutive viscosity models: one Newtonian and four non-Newtonian^62^, where a wide range of bulk shear rates *γ*_0_ = 50–1050 s^−1^ were examined. All the non-Newtonian models have similar viscosity predictions at low *γ*_0_, while the viscosity of the PL model becomes inaccurate at high *γ*_0_ region. Besides, we also found negligible difference in *γ*_max_ for water, blood as a Newtonian fluid, and blood as a non-Newtonian fluid (Fig. 5B). Interestingly, qualitative similarities between water and blood suggest that blood can be considered Newtonian in CFD analyses of shear rate distribution, even in low *γ*_0_ conditions (Fig. 5B).

This finding supports the feasibility of CFD practices with the water Newtonian model for microfluidic characterization as a reductionist approach with much reduced computational costs. Having said that, the water Newtonian model does not fully recapitulate the behavior of whole blood^61^. Viscoelastic models, such as the simplified Phan-Thien-Tanner or Gisekus^61^, are good alternatives to capture the blood rheology in future studies.

## Discussion

Our numerical studies present a refined CFD approach to map hemodynamic parameters for both concentric and eccentric stenosis microfluidic models of thrombosis, providing unprecedented rheological insights underlying biomechanical platelet aggregation and thrombosis. The results present that (1) the stenosis level *S* is the major determinant of the shear rate *γ* and shear rate gradient *γ*^′^ within disturbed flow at the micro-contraction; (2) the contraction angle *α* plays a significant role in governing *γ*^′^, while having negligible influence on *γ*_max_; (3) the shear rate *γ* experienced by washed platelets in Tyrode’s buffer is similar to that in whole blood; (4) water as a Newtonian fluid can be applied to microfluidic CFD characterization as a reductionist approach with reduced computational costs.

Previous experimental studies have demonstrated that in vivo aggregation is sensitive to *γ*^′^ at stenosis^7^. The follow-up studies suggested that *γ*_0_, *S* and *α* affect platelet aggregation in terms of kinetics, stability and sizes of aggregates^38^. The underlying platelet mechanobiology at molecular and cellular levels is exciting yet incompletely understood^5^. One possibility is that a *γ*^′^ threshold exists for VWF elongation, and its subsequent conformational activation^6,13,60,64^. The other working mechanism is due to the collision and compression between RBCs and platelets at the stenosis and surface of developing aggregates^16,41^. To investigate these mechanobiological mechanisms, future experiments are required to correlate our CFD results with molecular and cellular behaviors of biomechanical platelet aggregation in these stenotic microfluidic devices^13^.

## Supplementary Information

Supplementary Information

## Data Availability

The data that supports the findings of this study are available from the corresponding author upon reasonable request.

## References

[1] Virani SS (2020). Heart disease and stroke statistics-2020 update: a report from the american heart association. Circulation.

[2] Jackson SP (2011). Arterial thrombosis–insidious, unpredictable and deadly. Nat. Med..

[3] Jackson SP, Nesbitt WS, Westein E (2009). Dynamics of platelet thrombus formation. J. Thromb. Haemost.

[4] Rana A, Westein E, Niego B, Hagemeyer CE (2019). Shear-dependent platelet aggregation: mechanisms and therapeutic opportunities. Front. Cardiovasc. Med..

[5] Chen Y, Ju LA (2020). Biomechanical thrombosis: the dark side of force and dawn of mechano-medicine. Stroke Vasc. Neurol..

[6] Brazilek RJ (2017). Application of a strain rate gradient microfluidic device to von Willebrand's disease screening. Lab. Chip.

[7] Nesbitt WS (2009). A shear gradient-dependent platelet aggregation mechanism drives thrombus formation. Nat. Med..

[8] Schneider SW (2007). Shear-induced unfolding triggers adhesion of von Willebrand factor fibers. Proc. Natl. Acad. Sci. USA.

[9] Fu H (2017). Flow-induced elongation of von Willebrand factor precedes tension-dependent activation. Nat. Commun..

[10] Ju L, Lou J, Chen Y, Li Z, Zhu C (2015). Force-induced unfolding of leucine-rich repeats of glycoprotein ibalpha strengthens ligand interaction. Biophys. J..

[11] Zhang W (2015). Identification of a juxtamembrane mechanosensitive domain in the platelet mechanosensor glycoprotein Ib-IX complex. Blood.

[12] Ju L, Chen Y, Xue L, Du X, Zhu C (2016). Cooperative unfolding of distinctive mechanoreceptor domains transduces force into signals. Elife.

[13] Chen Y (2019). An integrin alphaIIbbeta3 intermediate affinity state mediates biomechanical platelet aggregation. Nat. Mater..

[14] Ting LH (2019). Contractile forces in platelet aggregates under microfluidic shear gradients reflect platelet inhibition and bleeding risk. Nat. Commun..

[15] Tovar-Lopez FJ (2011). Structural and hydrodynamic simulation of an acute stenosis-dependent thrombosis model in mice. J. Biomech..

[16] Tovar-Lopez FJ (2013). An investigation on platelet transport during thrombus formation at micro-scale stenosis. PLoS ONE.

[17] Hong JK (2020). Evaluating medical device and material thrombosis under flow: current and emerging technologies. Biomater. Sci..

[18] Kroll MH, Hellums JD, McIntire LV, Schafer AI, Moake JL (1996). Platelets and shear stress. Blood.

[19] Farag MB (2014). Review of recent results using computational fluid dynamics simulations in patients receiving mechanical assist devices for end-stage heart failure. Methodist. Debakey Cardiovasc. J..

[20] Nascimbene A, Neelamegham S, Frazier OH, Moake JL, Deong J (2016). Acquired von Willebrand syndrome associated with left ventricular assist device. Blood.

[21] Ballermann BJ, Dardik A, Eng E, Liu A (1998). Shear stress and the endothelium. Kidney Int..

[22] Papaioannou TG, Stefanadis C (2005). Vascular wall shear stress: basic principles and methods. Hellen. J. Cardiol..

[23] Dhawan SS (2010). Shear stress and plaque development. Expert Rev. Cardiovasc. Ther..

[24] Xing R (2018). Temporal and spatial changes in wall shear stress during atherosclerotic plaque progression in mice. R. Soc. Open Sci..

[25] Kim D, Bresette C, Liu Z, Ku DN (2019). Occlusive thrombosis in arteries. *APL*. Bioengineering.

[26] Mongrain R, Rodés-Cabau J (2006). Role of shear stress in atherosclerosis and restenosis after coronary stent implantation. Rev. Esp. Cardiol..

[27] Foin N (2014). Incomplete stent apposition causes high shear flow disturbances and delay in neointimal coverage as a function of strut to wall detachment distance. Circ. Cardiovasc. Interven..

[28] Piper R (2018). The mechanistic causes of peripheral intravenous catheter failure based on a parametric computational study. Sci. Rep..

[29] Lucas TC (2013). Blood flow in hemodialysis catheters: a numerical simulation and microscopic analysis of in vivo-formed fibrin. Artif. Organs.

[30] Yang S (2017). Computational simulation of postoperative pulmonary flow distribution in Alagille patients with peripheral pulmonary artery stenosis. Congenit. Heart Dis..

[31] Jin C, Liu Y (2019). Influence of competitive flow caused by different stenosis on coronary artery bypass hemodynamics and PIV study. Mol. Cell. Biomech..

[32] Zhao Z, Mao B, Liu Y, Yang H, Chen Y (2018). The study of the graft hemodynamics with different instant patency in coronary artery bypassing grafting. Comput. Model. Eng. Sci..

[33] Zhu C, Chen Y, Ju LA (2019). Dynamic bonds and their roles in mechanosensing. Curr. Opin. Chem. Biol..

[34] Zhao YC (2021). Computational fluid dynamics simulations at micro-scale stenosis for microfluidic thrombosis model characterization. Mol. Cell. Biomech..

[35] Zhou F, Chen Y, Felner EI, Zhu C, Lu H (2018). Microfluidic auto-alignment of protein patterns for dissecting multi-receptor crosstalk in platelets. Lab. Chip.

[36] Colace TV, Tormoen GW, McCarty OJ, Diamond SL (2013). Microfluidics and coagulation biology. Ann. Rev. Biomed. Eng..

[37] Hansen CE, Lam WA (2017). Clinical implications of single-cell microfluidic devices for hematological disorders. Anal. Chem..

[38] Tovar-Lopez FJ (2010). A microfluidics device to monitor platelet aggregation dynamics in response to strain rate micro-gradients in flowing blood. Lab. Chip.

[39] Sundd P (2012). ‘Slings’ enable neutrophil rolling at high shear. Nature.

[40] Tsai M (2012). In vitro modeling of the microvascular occlusion and thrombosis that occur in hematologic diseases using microfluidic technology. J. Clin. Invest..

[41] Ju L (2018). Compression force sensing regulates integrin alphaIIbbeta3 adhesive function on diabetic platelets. Nat. Commun..

[42] Cho YI, Kensey KR (1991). Effects of the non-Newtonian viscosity of blood on flows in a diseased arterial vessel. Part 1: Steady flows. Biorheology.

[43] Abraham F, Behr M, Heinkenschloss M (2005). Shape optimization in steady blood flow: a numerical study of non-Newtonian effects. Comput. Methods Biomech. Biomed. Engin..

[44] Sharma K, Bhat S (1992). Non-Newtonian rheology of leukemic blood and plasma: are n and k parameters of power law model diagnostic?. Physiol. Chem. Phys. Med. NMR.

[45] Ballyk P, Steinman D, Ethier C (1994). Simulation of non-Newtonian blood flow in an end-to-side anastomosis. Biorheology.

[46] Fung Y-C (2013). Biomechanics: mechanical properties of living tissues.

[47] Walburn FJ, Schneck DJ (1976). A constitutive equation for whole human blood. Biorheology.

[48] Soulis JV (2008). Non-Newtonian models for molecular viscosity and wall shear stress in a 3D reconstructed human left coronary artery. Med. Eng. Phys..

[49] Miller C (1972). Predicting non-Newtonian flow behavior in ducts of unusual cross section. Ind. Eng. Chem. Fundam..

[50] Lancellotti RM, Vergara C, Valdettaro L, Bose S, Quarteroni A (2017). Large eddy simulations for blood dynamics in realistic stenotic carotids. Int. J. Numer. Method Biomed. Eng..

[51] Lee SE, Lee SW, Fischer PF, Bassiouny HS, Loth F (2008). Direct numerical simulation of transitional flow in a stenosed carotid bifurcation. J. Biomech..

[52] Stroud JS, Berger SA, Saloner D (2002). Numerical analysis of flow through a severely stenotic carotid artery bifurcation. J. Biomech. Eng..

[53] Fox RW, McDonald AT, Mitchell JW (2020). Fox and McDonald's introduction to fluid mechanics.

[54] Vahidkhah K, Balogh P, Bagchi P (2016). Flow of red blood cells in stenosed microvessels. Sci. Rep..

[55] Fedosov DA, Caswell B, Popel AS, Karniadakis GE (2010). Blood flow and cell-free layer in microvessels. Microcirculation.

[56] Fung Y-C (2013). Biomechanics: motion, flow, stress, and growth.

[57] Wurzinger L, Opitz R, Wolf M, Schmid-Schönbein H (1985). “Shear induced platelet activation”-A critical reappraisal. Biorheology.

[58] Hellums, J., Peterson, D., Stathopoulos, N., Moake, J. & Giorgio, T. in *Cerebral ischemia and hemorheology* 80–89 (Springer, 1987).

[59] O'Brien J (1990). Shear-induced platelet aggregation. The Lancet.

[60] Ju L, Dong JF, Cruz MA, Zhu C (2013). The N-terminal flanking region of the A1 domain regulates the force-dependent binding of von Willebrand factor to platelet glycoprotein Ibalpha. J. Biol. Chem..

[61] Campo-Deaño L, Dullens RP, Aarts DG, Pinho FT, Oliveira MS (2013). Viscoelasticity of blood and viscoelastic blood analogues for use in polydymethylsiloxane in vitro models of the circulatory system. Biomicrofluidics.

[62] Mendieta JB (2020). The importance of blood rheology in patient-specific computational fluid dynamics simulation of stenotic carotid arteries. Biomech. Model. Mechanobiol..

[63] Brust M (2013). Rheology of human blood plasma: viscoelastic versus Newtonian behavior. Phys. Rev. Lett..

[64] Momi S (2013). Reperfusion of cerebral artery thrombosis by the GPIb-VWF blockade with the Nanobody ALX-0081 reduces brain infarct size in guinea pigs. Blood.

