# Supplementary material for: Hemodynamic analysis for stenosis microfluidic model of thrombosis with refined computational fluid dynamics simulation
Source: Sci Rep. 2021 Mar 25;11:6875. doi: 10.1038/s41598-021-86310-2 (PMC7994556; doi:10.1038/s41598-021-86310-2)
Supplement: Supplementary file 1 — Supplementary Information [file 41598_2021_86310_MOESM1_ESM.docx]

**Supplementary information**

**Title: Hemodynamic analysis for stenosis microfluidic model of thrombosis with refined computational fluid dynamics simulation**

**Yunduo Charles Zhao^1,2†^, Parham Vatankhah^1†^, Tiffany Goh^1,2,3^, Rhys Michelis^4^, Kiarash Kyanian^1^, Yingqi Zhang^1^, Zhiyong Li^5^ and Lining Arnold Ju^1,2,3*^**

^1^School of Biomedical Engineering, Faculty of Engineering, The University of Sydney, Darlington, NSW Australia 2008

^2^Charles Perkins Centre, The University of Sydney, Camperdown, NSW Australia 2006

^3^Heart Research Institute, Newtown, NSW, Australia 2042

^4^School of Chemical and Biomolecular Engineering, Faculty of Engineering, The University of Sydney, Darlington, NSW Australia 2008

^5^School of Mechanical, Medical and Process Engineering, Queensland University of Technology, Brisbane Australia 4000

^*^corresponding author: arnold.ju@sydney.edu.au
**^†^**these authors contributed equally to this work

**Mesh and convergence criteria sensitivity verification**

In order to examine the sensitivity of the numerical results to our mesh size and applied convergence criteria (the root mean square error of approximation between two successive iterations), a thorough sensitivity verification study was conducted. To quantitatively measure mesh sensitivity, we examined the shear rate and velocity profile along a vertical sample line in the *y*-axis (Supplementary Fig. S1A). The sample line (20µm high) is located at the center of microchannel which connects the apex of stenosis area to the ceiling of the microchannel. To investigate the sensitivity of the results to the different mesh sizes, three studies with total number of 1,005,040 (*Fine*), 2,010,840 (*Finer*) and 2,868,187 (*Finest*) computational cells were studied for *γ*_0_ = 50 s^-1^ (Supplementary Fig. S1B) and *γ*_0_ = 3,000 s^-1^ (Supplementary Fig. S1C). Our numerical results showed the velocity and shear rate profiles for the 3 mesh sizes are superimposed, indicating that the simulation outcome is independent of the mesh size selection. Furthermore, studies were conducted to analyze the convergence criteria sensitivity, examining 1e^-3^, 1e^-6^ and 1e^-9^ convergence criteria under *γ*_0_ = 1,000 s^-1^ (Supplementary Fig. S1D). The simulated velocity profiles are also superimposed, suggesting the results are independent of the applied convergence criteria. It is worth noting that all the simulations in this study were performed using the *finer* grid and 1e^-6^ convergence criteria.


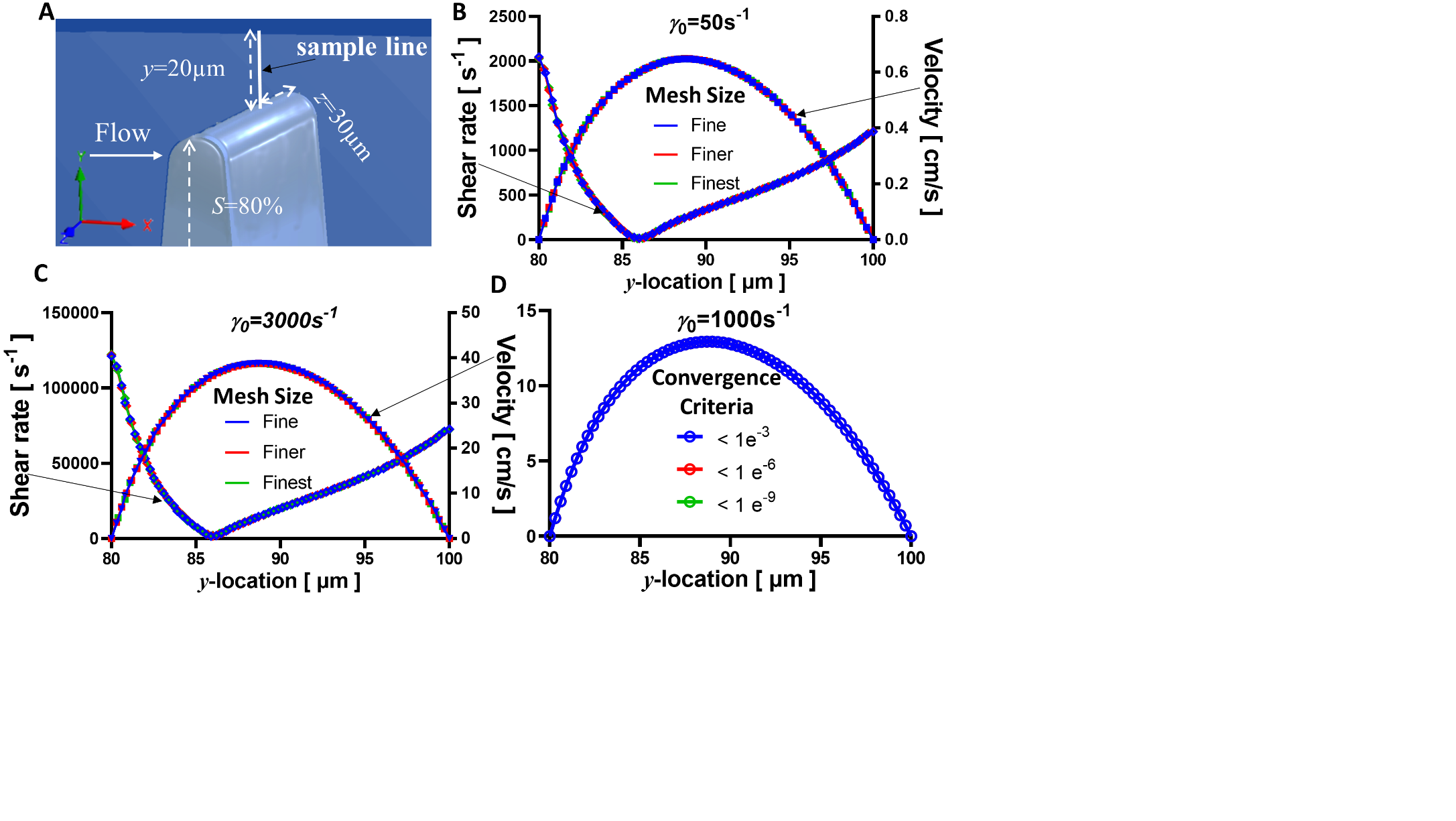


**Supplementary Figure S1. CFD simulated mesh and convergence criteria sensitivity verification.** (A) The vertical sample line of the sensitivity verification study, which locates 30µm height to the bottom in the *z*-direction and connects 20µm spacing between the ceiling and the surface of stenosis apex along *y*-direction. **(**B and C) Shear rate and velocity profiles along the sample line under *γ*_0_=50 s^-1^ (B) and *γ*_0_=3,000 s^-1^ (C) with 3 different meshing sizes. Note the 3 lines are superimposed with each other. (D) The velocity distribution along the sample line under *γ*_0_=1,000 s^-1^ with 3 convergence criteria of < 1e^-3^, < 1e^-6^ and < 1e^-9^.

**Analytical validation for GPL model at high shear rate**

For complex geometries such as the stenosis microfluidic, analytical solutions for Generalized Power-Law GPL models are unavailable. In this context, we employed a cylindrical microchannel (200µm diameter and 800µm long) to test if GPL returns reliable results compared with the analytical solution (Supplementary Fig. S2A). The bulk shear rate *γ*^­^_0_=3,000 s^-1^ was examined where the non-Newtonian behavior diminishes (Fig. 5A). Besides, we also compared the velocity profile of the non-Newtonian and Newtonian model in the fully developed region (Supplementary Fig. S2B).

The velocity profile of laminar flow in a cylindrical microchannel is expressed as

$u\left( r \right)=2u_{m}[1-\left( \frac{r}{r_{0}} \right)^{2}]$ (S1)

where $u\left( r \right)$ is the velocity profile (m s^-1^), $u_{m}$ the mean velocity (m s^-1^), $r$ is the radial distance from the center of tube (m), and $r_{0}$ is the microchannel’s radius (m).

The bulk shear rate *γ*^­^_0_ is then calculated by Supplementary Eq. S2.

$\gamma_{0}=\frac{4u_{m}}{r_{0}}$ (S2)

The fully developed velocity profile and the wall shear rate is calculated numerically for the three grids and compared to the analytical solution for the Newtonian fluid (Supplementary Fig. S2A and B). The results demonstrate superimposed shear rate and velocity profiles, suggesting that the numerical simulated GPL model has the same behavior as the analytical solution at high shear rate conditions.


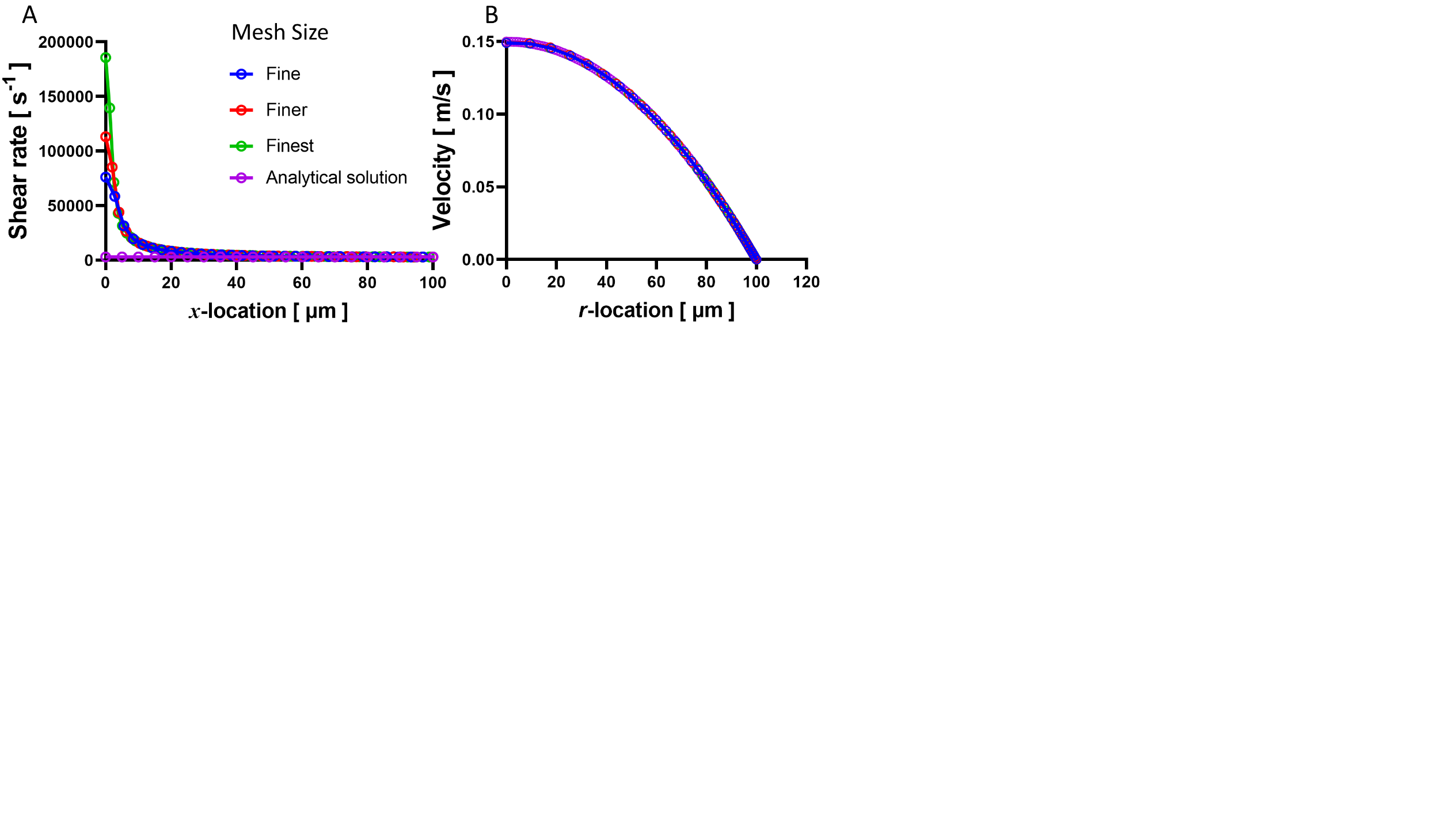


**Supplementary Figure S2. Validation analysis of shear rate and velocity for GPL model**. A) CFD simulated shear rate distribution utilizing GPL model vs. the analytical solution. Note 3 mesh sizes were examined with the total number of $34\times136$, $48\times160$, and $60\times200$ computational cells, named fine, finer and finest, respectively. B) Velocity distribution for 3 different mesh sizes and analytical solution as aforementioned. Note that the Finer (Red) and Finest (Green) lines are nearly invisible.
